# Kinematic and Kinetic Analysis of Two Gymnastics Acrobatic Series to Performing the Backward Stretched Somersault

**DOI:** 10.2478/hukin-2013-0021

**Published:** 2013-07-05

**Authors:** Bessem Mkaouer, Monèm Jemni, Samiha Amara, Helmi Chaabène, Zouhair Tabka

**Affiliations:** 1 Higher Institute of Sport and Physical Education of Ksar Saïd, Univercity of Manouba, Tunisia.; 2 School of Science, University of Greenwich, London, United Kingdom.; 3 Faculty of Medicine of Sousse, University of Sousse, Tunisia.

**Keywords:** motion analysis, take-off, flic-flac, tempo salto

## Abstract

Back swing connections during gymnastics acrobatic series considerably influence technical performance and difficulties, particularly in the back somersault. The aim of this study was to compare the take-off’s kinetic and kinematic variables between two acrobatic series leading to perform the backward stretched somersault (also called salto): round-off, flic-flac to stretched salto versus round-off, tempo-salto to stretched salto. Five high level male gymnasts (age 23.17 ± 1.61 yrs; body height 1.65 ± 0.05 m; body mass 56.80 ± 7.66 kg) took part in this investigation. A force plate synchronized with a two dimensional movement analysis system was used to collect kinetic and kinematic data. Statistical analysis via the non-parametric Wilcoxon Rank-sum test showed significant differences between the take-offs’ variables. The backswing connections were different in the take-off angle, linear momentum, vertical velocity and horizontal and vertical displacements. In conclusion, considering that the higher elevation of the centre of mass in the flight phase would allow best performance and lower the risk of falls, particularly when combined to a great angular momentum, this study demonstrated that the optimal connection series was round-off, flic-flac to stretched salto which enabled the best height in the somersault. Analysis of the results suggests that both connections facilitate the performance of single and double (or triple) backward somersaults with or without rotations around the longitudinal axis. Gymnasts could perform these later while gaining height if they chose the round-off, flic-flac technique or gaining some backward displacement if they choose the round-off, salto tempo.

## Introduction

In accordance with the mechanical laws, the take-off’s characteristics (arm swing, leg impulse and velocity of backward displacement) determine both angular momentum, trajectory of the centre of mass (COM) and total flight time of a gymnast during acrobatic aerial flight ( McNitt-Gray et al., 1994 ; Sands, 2011; Smith, 1983 ). Generally speaking, the somersault results from the coordinated involvement of body parts that is imposed to generate an optimal solution to constraints occurring during the execution (whether external constraint (such as gravity) or internal ones (such as the relative orientation of body segments and the inertial characteristics of these segments)). This requires an optimal force and velocity that are related to the gymnast’s ability to create sufficient momentum enabling body management during rotations ( Bardy and Laurent, 1994 ; McNitt-Gray, 2001 ; McNitt-Gray et al., 2006 ).

In gymnastics, the most difficult acrobatic movements on the floor depend upon the efficient execution of the transitional skills, i.e. the roundoff, flic-flac and/or tempo-salto acting as accelerators prior to the take-off. The backward take-off initiates the linear and rotational impulses for somersaults with various body positions (tucked, picked, straight or stretched). The aim of the take-off that precedes a stretched backward somersault on the floor routine is the optimization of the associated variables: i.e. the velocity by attaining a large amount of kinetic energy necessary to achieve a large angular momentum’s magnitude. So far, the most comprehensive studies on backward take-offs have been provided by Brüeggemann (1983 , 1994 ), Geiblinger et al. (1995) , Hwang et al. (1990) , Knoll (1992) , Knoll and Krug (1990) , Mkaouer et al. (2012) and Sadowski et al. (2005) . The authors outlined key components of the back somersault performed after round-off, flic-flac and they agreed that a speed of 5 to 6 m/s and a take-off angle between 75 and 85° were optimal to perform the backward stretched somersault.

In artistic gymnastics, the transition skills are decisive to successfully and safely perform acrobatic elements. A gymnast must obtain the required quantity of movement at the end of this phase in order to guarantee optimal linear and rotational momentums enabling enough centre of mass’ elevation for a full 360° straight body aerial rotation during a somersault. Gravity is the only force acting on the gymnast during the flight period of a somersault. The main consequence is that the angular momentum is constant between the take-off and landing (based on the principle of conservation of angular momentum). In this respect, the choice of the technical preparatory backswing elements allowing the somersault’s take-off is crucial for optimal performance.

The purpose of this investigation was to compare the take-off’s kinetic and kinematic variables between two acrobatic series leading to perform the backward stretched somersault (also called salto): round-off, flic-flac to stretched salto versus round-off, tempo-salto to stretched salto.

## Material and Methods

### Participants

Five elite male gymnasts (age 23.17±1.61 yrs; body height 1.65±0.05 m; body mass 56.80±7.66 kg) volunteered to take part in this study. The inclusion criteria were: to be ranked at international level with participation in world cups and/or championships; average training volume around 25 hours per week; healthy without any muscular, neurological or tendinitis injuries; able to perform the two acrobatic series. After being informed about the procedures, methods, benefits and possible risks involved in the study, each subject reviewed and signed a consent form to participate in the study. The experimental protocol was performed in accordance with the Declaration of Helsinki for human experimentation and was approved by the university of Manouba ethical committee.

### Measurements

This research was a simultaneous dual approach study (kinematic and dynamic) of two acrobatic backswing connection series: round-off, flic-flac vs round-off tempo-salto leading to performing backward stretched salto. These two connections were different in the arms and snap down actions: the first series (round-off, flic-flac to stretched backward salto (RFS)) was performed with hands’ push-off and a large range of motion (ROM) in the snap down ( Figure 1a ); the second series (round-off, salto-tempo to stretched backward salto (RTS)), was performed without hands’ push-off and with a medium ROM in the snap down ( Figure 1b ).

Take-off’s direct kinetic data of the back somersaults were measured using a Kistler Quattro jump force plate (ref. 2822A1-1, sampling frequency 500 Hz, size 100 × 100 × 12 cm) and analyzed using a Quattro Jump Bosco Protocol Software 1.0.9.2 (Kistler Instruments, Switzerland). Maximal vertical force (Fy _max_ ) and maximal rate of force development (RFD _max_ ) were analysed following the data acquisition.

In order to collect kinematic data, twenty retro-reflective body markers were attached to the gymnasts’ bodies in order to enable digitation. The salto sequences were recorded using two cameras (50 Hz; Sony DCR PC108E Mini DV, 1 million pixels CCD and SSC 1/4000 per second) with wide conversion lens (× 0.6; 45.5 × 29 mm). Body markers were digitized using a video based data analysis system (SkillSpector 1.3.2) ( Nicolas and Bideau, 2009 ; Halawish, 2011; Bini et al., 2012 ; Sinclar et al., 2012 ). The body segments’ centres of mass were computed using the De Leva (1996) model. The centre of mass’ displacement (COM dx and dy) and velocity (COM vx and vy) were analysed. The angular data of the shoulder (α s ), hip (α h ) and knee joint angles (α k ) during the take-off were also analysed. The angular displacements of the same joints (θ s , θ h and θ k respectively ) and their angular velocities (ω s , ω h and ω k ) were calculated in the sagittal plane. In addition, the take-off’s angle (α t ) was calculated using the freeware MB-Ruler version 5.0.

The indirect kinetic data: linear momentum of the upper limbs (px _ul_ and py _ul_ ), lower limbs (px _ll_ and py _ll_ ) and trunk (px _tc_ and py _tc_ ) were studied following data acquisition.

### Procedures

Testing was carried out in the Gymnasium of the Olympic City within a 3-day period, starting at 4:00 pm up to 6:00 pm under the following environmental conditions: average temperature 23°C (minimum 20, maximum 26°C). The force plate was integrated into the extremity of the acrobatic track and synchronized with the two cameras. The first camera was placed in front at 3m and the second sideways at 7m from the acrobatic track. During all procedures, the participants wore only shorts and gymnastic sneakers. A 15-minute warm-up, based on light jogging, stretching and several easy acrobatic elements was allowed before testing.

Each gymnast started in a standing position at the start of the acrobatic track. He was required to randomly perform one of the acrobatic series at a precise signal. Three attempts were required for each of acrobatic series (roundoff, flic-flac, backward stretched salto and/or round-off, salto-tempo, backward stretched salto). The execution of each acrobatic series was separated by two minutes of recovery and a five-minute rest period between the two techniques. Only the best somersault of each acrobatic series was retained for the comparative study. An experienced international competition judge marked all trials and helped to choose the best somersaults to be considered for analysis.

### Analysis

Data are reported as mean ± standard deviation (SD). Effect size (dz) was calculated using GPOWER software (Bonn FRG, Bonn University, Department of Psychology) ( Faul and Erdfelder, 2004 ). The following scale was used for the interpretation of *d* z: < 0.2, [trivial]; 0.2–0.6, [small]; 0.6–1.2, [moderate]; 1.2–2.0, [large]; and >2.0, [very large] ( Scanlan et al., 2012 ). The normality of distribution estimated by the Kolmogorov-Smirnov test was not acceptable for all variables. Therefore, the non-parametric Wilcoxon Rank-sum test was applied to compare the acrobatic series pair-wise. The results were considered significantly different when the probability was less than or equal to 0.05 ( *p* ≤ 0.05 ). Statistical analyses were performed using the software package SPSS version 13.0 (SPSS Inc., Chicago, IL, USA).

## Results

Table 1 shows all the descriptive kinetic and kinematic variables. These were compared between the two acrobatic series and presented in Table 2 . The Wilcoxon Rank-sum Test demonstrated that the two acrobatic series (RFS and RTS) had different effect on the backward stretched salto. The following paragraphs highlight the main findings:

All direct kinetic data (Fy _max_ and RFD _max_ ) were almost similar between RFS and RTS during the take-off phase. Moreover, indirect kinetic data showed a considerable difference between the acrobatic series. The linear momentum’s horizontal component of the lower limbs (px _ll_ ) was increased by 81.96% in the RFS with respect to RTS ( *p <* 0.05 ). Similarly, the linear momentum’s vertical component of the upper limbs (py _ul_ ) was increased by 25.69% ( *p <* 0.05 ) in favour of the RFS. Also, the horizontal component (px _ul_ ) was increased by 31.01% in the RTS with respect to RFS ( *p <* 0.05 ) and the linear momentum’s vertical component of the trunk (py _tc_ ) was significantly increased by 88.29% in the same condition ( *p <* 0.05 ) ( Table 2 ).

Interestingly, the linear momentum’s vertical component of the lower limbs (py _ll_ ) and the linear momentum’s horizontal component of the trunk (px _tc_ ) did not differ between both conditions. With regards to the kinematic data, the take-off angle (α t ) was decreased in the RFS series with respect to RTS: (Δ = – 12.07% with *p <* 0.05 ). Moreover, the shoulder joint’s angle at the take-off (α s ) was increased by 35.48% ( *p <* 0.05 ) and the hip joint’s angular displacement (θ h ) was increased by 30.23% ( *p <* 0.05 ). The hip joint’s angle at the take-off (α h ) was increased in RTS series with respect to RFS: (Δ = 19.88% with *p <* 0.05 ). Likewise, the angular velocity of the hip joint (ω h ): (Δ = 21.93% with *p <* 0.05 ) and the horizontal displacement of the COM (dx): (Δ = 8.39% with *p <* 0.05 ) were all increased. Moreover, the vertical displacement of the COM (dy) was decreased in RTS with respect to RFS: (Δ = 37.09% with *p <* 0.05 ) and the same was observed for the vertical velocity (vy): (Δ = 20.62% with *p <* 0.05 ). The angular velocity of the shoulder joint (ω s ) and the knee joint (ω k ) did not vary during the different backswings connection series. In the same way, the knee joint’s angle (α k ), the angular displacement at the shoulder joint (θ s ) and the knee joint (θ k ) remained almost identical during the take-off. Finally, the horizontal velocity of the COM (vx) was approximately equal ( Table 1 ).

## Discussion

Two crucial biomechanical criteria are considered when assessing the technical performance of backswing connection in back acrobatic series: vertical velocity at the take-off and vertical elevation of the gymnast’s centre of mass during the aerial phase of the somersault. With a better velocity and elevation of the COM, the stability of landing is much more secured, particularly when combined with longitudinal rotations (twists).

This study is focused on the variables that could affect take-off phases by comparing them between two different acrobatic series. The different backswing connection did not affect the direct kinetic data at the take-off during the stretched back somersault. The vertical force and the maximal rate of force development remained almost identical. Moreover, the indirect kinetic data showed a significant difference ( *p* < 0.05 ). The RTS connection allowed a larger linear momentum of the trunk on the vertical axis and the upper limbs on the horizontal axis ( Figure 2a and 2b ). Similarly, the RFS connection allowed a larger linear momentum of the upper limbs on the vertical axis and the lower limbs on the horizontal axis ( Figure 2c and 2d ). These results were in accordance with those of Hraski and Mejovsek (2004) .

This difference in body parts’s linear momentum could be explained by the nature of the snap down and the contribution of the arms in the entire motion. The snap down was closer to the vertical axis during the RTS connection series when compared to the RFS ( Figure 3a and 3b ). Similarly, arms’ action in RFS was more important than in RTS in the vertical axis. Also, the difference between RFS and RTS could be explained by the difficulty of the tempo-salto, it was considered as an element of higher difficulty “B” equivalent to the backward stretched salto compared to the flic-flac that was classified as “A” difficulty according to the FIG Code of Points 2009 (International Gymnastics Federation).

In this study, the take-off’s angle (α t ) was significantly higher during the RFS than during the RTS ( *p <* 0.05 ). These results were in accordance to those presented by Hraski (2002) who found an angle of 80° and Geiblinger et al. (1995) who reported an angle of 88 ± 3.0° for the backward stretched salto. Similarly, according to Sadowski et al. (2005) , the take-off angle was different between gymnasts but was within a range of 7° forward of the vertical line and 5° backward. We have to remind that the angle of the shoulder joint (α s ) showed a greater opening in the RFS series vs RTS in the present study ( *p <* 0.05 ). In addition, the angle of the hip joint (α h ) was wider in the RTS vs RFS ( *p <* 0.05 ) ( Figure 3a and 3b ). These joints’ angles were relatively similar to those reported by Boloban et al. (2007) , Huang and Hsu (2009) , and Sadowski et al. ( 2005 and 2009 ).

The horizontal velocity of the COM at the take-off was comparable in all acrobatic series. However, it varied for the vertical velocity: the connection RFS displayed significantly higher values ( *p* < 0.05 ) than the RTS. Furthermore, the angular velocity of the hip (ω h ) was larger at RTS, but the angular displacement of the hip ( 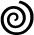 H) was more reduced compared to the RFS ( *p* < 0.05 ). The overall outcomes were similar to those reported by Sadowski et al. (2009) , Kerwin et al. (1998) , Hwang et al. (1990) and Brüggemann (1983) (4.60, 4.50, 4.46 and 4.57 m / s respectively).

When the gymnast was leaving the floor, we noted that different connections affected the flight/aerial phase. The vertical and horizontal displacements of the centre of mass (COM) varied considerably ( *p* < 0.05 ). In the vertical axis, the maximum peak was reached in RFS; this could obviously allow the combination of multiple rotations around the transverse and the longitudinal axis during the salto/s (single, double or triple somersaults with twists). On the contrary, in the horizontal axis the maximum displacement was attained in the RTS series; this could similarly allow the combination of multiple rotations around the transverse and longitudinal axis during the salto/s (single, double or triple somersaults with twists). Depending on the physical fitness and power of the gymnast, each would have to choose the best compromise guaranteeing good technique and safe landing.

Those who are powerful enough to gain lots of height could perform their somersaults and twists within an ascendant and/or descendant phase and those who cannot go too high would compensate by a transversal displacement. Gymnastics coaches indicate that gymnasts could trigger the twists at any time of the flight. Some gymnasts trigger them immediately at the take-off, others in the middle after gaining some height and others at the end of the ascendant phase of the flight. The completion of the twist number could be affected by the trigging time. The results of the horizontal and vertical displacements were comparable to those reported by Sadowski et al. (2005) and Hraski (2002) (1.38 and 2.35 m; 1.25 and 2.52 m respectively). In addition, they were superior to the results of Cuk and Ferkolj (2000) and Geiblinger et al. (1995) (0.70 and 2.67 m; 0.86 m and 2.71 respectively).

Table 3 highlights the main kinetic and kinematic findings of this study.

## Conclusions

The aim of the study was to compare the mechanical effects of two backswing connection series (round-off, flic-flac vs round-off saltotempo) used in the preparation phase of the acrobatic series prior to the completion of the backward stretched salto. It ultimately aimed to identify the connection that resulted in a more efficient performance of the skill. The effects of the two different backswing connections were notorious on the backward stretched salto. The combination round-off, tempo-salto allowed greater horizontal displacement and momentum, while the combination round-off, flic-flac, salto allowed better vertical displacement and velocity. This difference in motion could be related to the type of the push-offs the gymnasts used in the snap down phase during the somersault. The direction of the reaction forces was different between the two series. It was thrown off centre forward but close to the centre of mass during the RFS and significantly thrown off the centre forward during the RTS. Finally, the connection round off flic-flac allowed better elevation of the gymnast’s centre of mass, however, each technique could provide specific benefits to the gymnasts: analysis of the results suggests that both connections facilitate the performance of single and double (or triple) backward somersaults with or without rotations around the longitudinal axis. Gymnasts could perform these later while gaining height if they choose the RFS technique or gaining some backward displacement if they select the RTS.

## Figures and Tables

**(Fig.1a):**
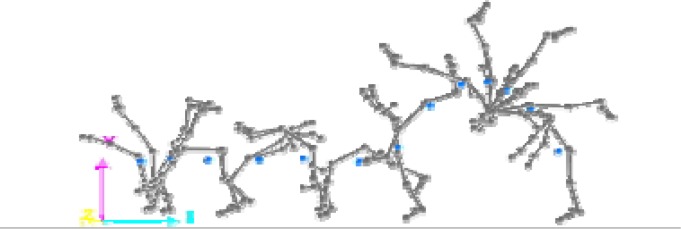
*
Round-off, flic-flac to stretched backward salto (RFS)
*

**(Fig. 1b):**
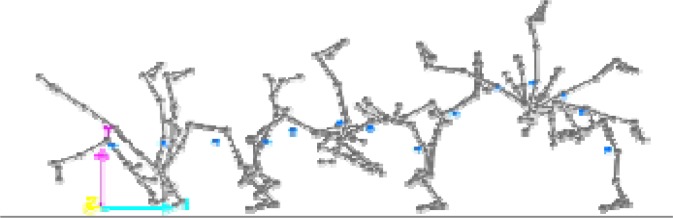
*
Round-off, salto-tempo to stretched backward salto (RTS)
*

**(Fig. 2a):**
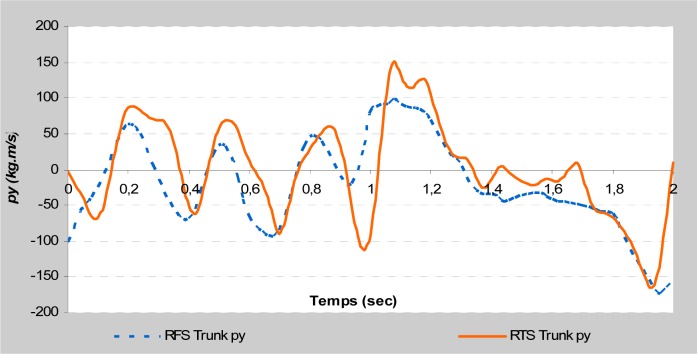
*
Vertical momentum of trunk
*

**(Fig. 2b):**
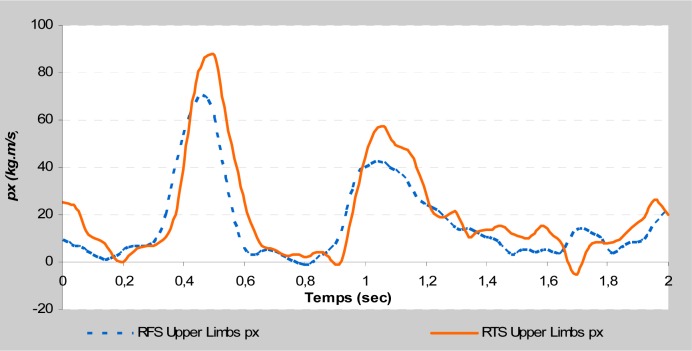
*
Horizontal momentum of upper limbs
*

**(Fig. 2c):**
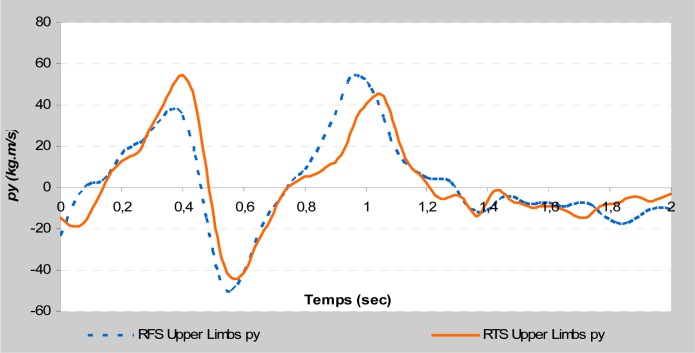
*
Vertical momentum of upper limbs
*

**(Fig. 2d):**
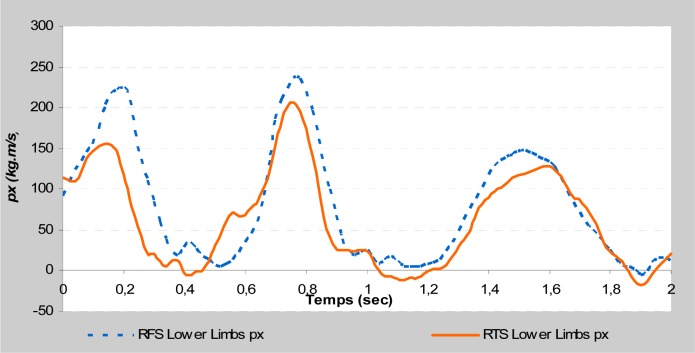
*
Horizontal momentum of lower limbs
*

**(Fig. 3a):**
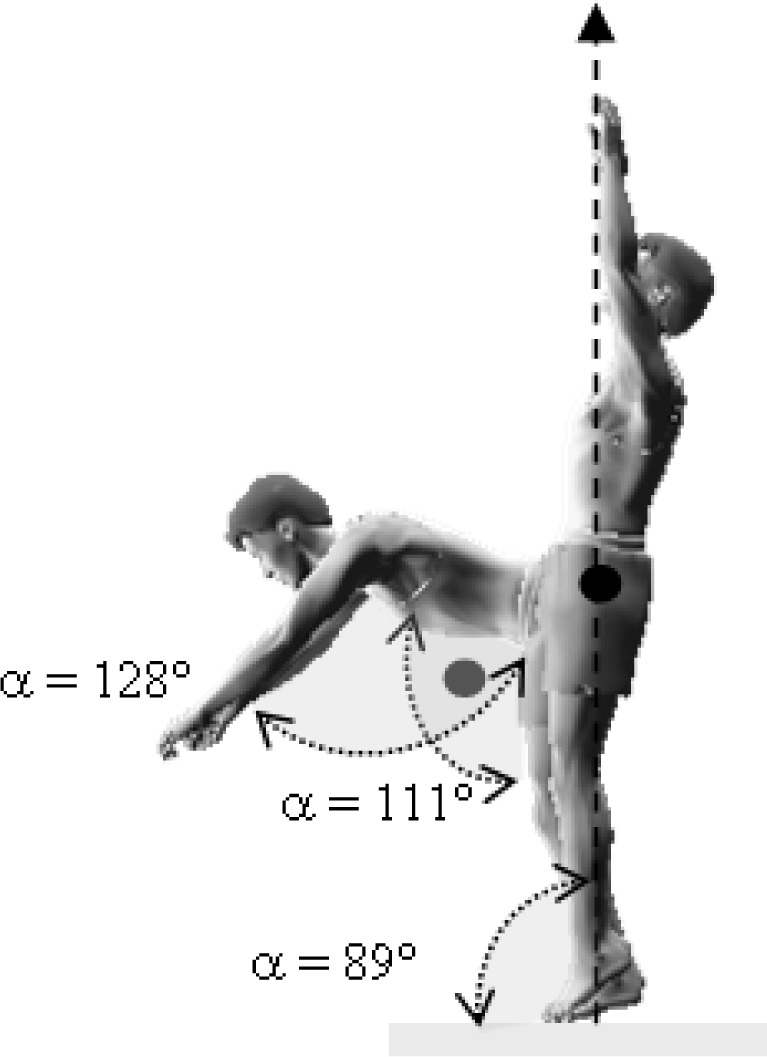
*
Round-off, flic-flac, salto backward stretched (RFS)
*

**(Fig. 3b):**
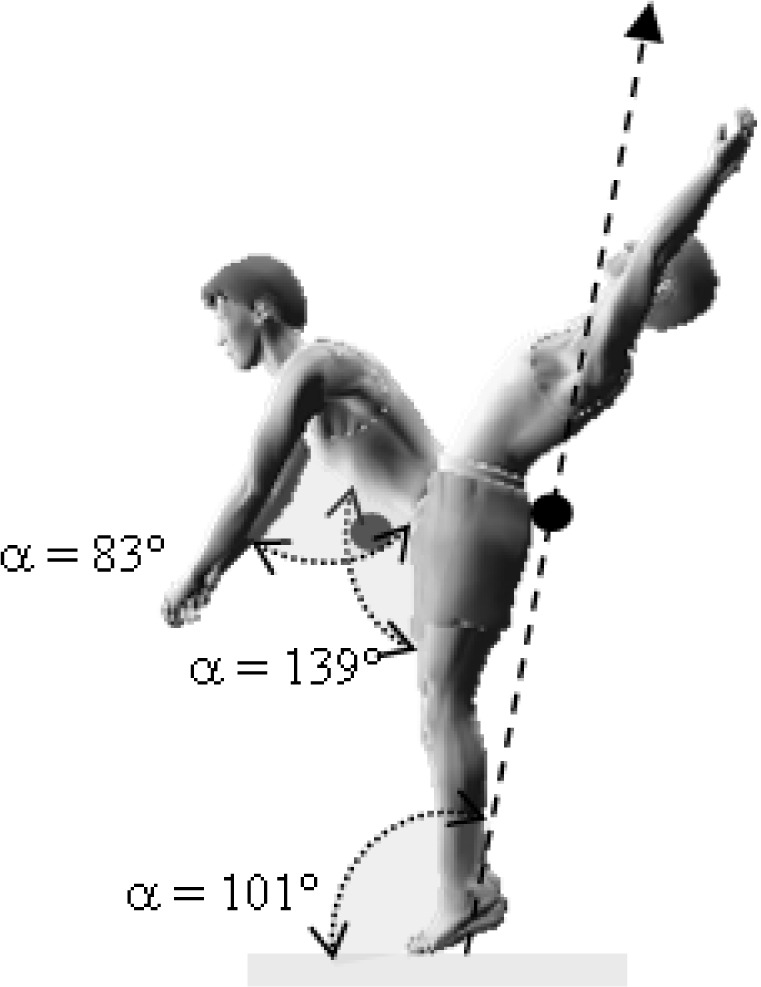
*
Round-off, salto-tempo, salto backward stretched (RTS)
*

**Table 1:** *
Descriptive statistic of backswing connections
*

** Variables **		** RFS **		** RTS **	
		Mean	SD	Mean	SD
** Kinetic **	Fy _ max _ (N)	6874.400	1204.708	8423.000	1427.718
	RDF _ max _ (N/s)	6829.772	2651.394	6439.628	507.692
	px _ ul _ (kg.m/s)	14.918	2.590	21.624	6.612
	py _ ul _ (kg.m/s)	42.824	2.872	31.819	4.671
	px _ ll _ (kg.m/s)	29.489	4.677	5.317	2.823
	py _ ll _ (kg.m/s)	124.044	8.684	121.117	7.403
	px _ tc _ (kg.m/s)	184.016	14.618	185.551	24.454
	py _ tc _ (kg.m/s)	11.095	3.065	94.782	20.623

** Kinematic **	dx (m)	2.598	0.149	2,836	0,317
	dy (m)	1.232	0.120	0.775	0.192
	vx (m/s)	3.743	0.367	3.513	0.808
	vy (m/s)	4.500	0.385	3.572	0.531
	α t (°)	89.04	1.54	101.26	3.69
	α s (°)	128.759	6.701	83.066	15.643
	α h (°)	111.585	8.165	139.288	8.611
	α k (°)	168.952	3.930	167.288	11.750
	θ s (°)	137.890	10.084	118.630	18.332
	θ h (°)	83.719	12.819	58.406	7.983
	θ k (°)	55.279	18.608	36.903	9.369
	ω s (°/s)	759.816	147.386	937.934	104.894
	ω h (°/s)	770.714	41.557	987.332	194.199
	ω k (°/s)	361.211	31.494	346.292	27.724

*(α): angle; (θ): angular displacement; (ω): angular velocity; (d): linear displacement; (T): take-off; (S): shoulder joint; (H): hip joint; (K): knee joint; (ul): upper limbs; (ll): lower limbs; (tc): trunk; (max): maximum; (X): horizontal component; (Y): vertical component; (F): force; (v): velocity; (RDF): rate of force development; (p): linear momentum.*

**Table 2:** *
Comparative analysis of backswing connections
*

** Variables **		** Wilcoxon Rank-sum Test **		** Effect size **
		Z	Sig.	* d * z
** Kinetic **	Fy _ max _ (N)	−1.483	0.138	---
	RDF _ max _ (N/s)	−0.135	0.893	---
	px _ ul _ (kg.m/s)	−2.023	0.043 *	1.162
	py _ ul _ (kg.m/s)	−2.023	0.043 *	2.214
	px _ ll _ (kg.m/s)	−2.023	0.043 *	5.315
	py _ ll _ (kg.m/s)	−0.674	0.500	---
	px _ tc _ (kg.m/s)	−0.674	0.500	---
	py _ tc _ (kg.m/s)	−2.023	0.043 *	4.013

** Kinematic **	dx (m)	−2.023	0.043 *	1.001
	dy (m)	−2.023	0.043 *	3.808
	vx (m/s)	−0.405	0.686	---
	vy (m/s)	−2.023	0.043 *	1.572
	α t (°)	−2.023	0.043 *	3.229
	α s (°)	−2.023	0.043 *	4.548
	α h (°)	−2.023	0.043 *	7.335
	α k (°)	−0.405	0.686	---
	θ s (°)	−1.753	0.08	---
	θ h (°)	−2.023	0.043 *	3.822
	θ k (°)	−1.483	0.138	---
	ω s (°/s)	−1.483	0.138	---
	ω h (°/s)	−2.023	0.043 *	1.113
	ω k (°/s)	−1.214	0.225	---

* *Significant at p < 0.05*

***d*** *z (sample size effect): < 0.2, [trivial]; 0.2–0.6, [small]; 0.6–1.2, [moderate]; 1.2–2.0, [large]; and >2.0, [very large]*
